# Knowledge and Attitudes Regarding Geriatric Depression: A Descriptive Study Among Adult Saudi Citizens

**DOI:** 10.7759/cureus.63797

**Published:** 2024-07-04

**Authors:** Ebtesam A Alzeiby, Hana A Alzuabi, Seba T Al-Gunaid, Bashayr Alkhalifah, Norah Bajunaid, Tamer M Hifnawy

**Affiliations:** 1 Department of Psychology, College of Education and Human Development, Princess Nourah bint Abdulrahman University, Riyadh, SAU; 2 College of Medicine, Princess Nourah bint Abdulrahman University, Riyadh, SAU; 3 College of Medicine, Syiah Kuala University, Banda Aceh, IDN; 4 Public Health And Community Medicine, Faculty of Medicine, Beni-Suef University, Beni-Suef, EGY

**Keywords:** geriatric care, geriatric depression, mental health, elderly, depression

## Abstract

Background: Depression is a leading cause of disability and contributes significantly to the overall burden of any disease. In Saudi Arabia, the geriatric population over 65 is continuing to expand and will contribute to a significant portion of the Saudi community. As the population of the elderly continues to grow as a result of longer lifespans, there will be an increase in the number of individuals in this population suffering from undiagnosed geriatric depression.

Aim of the work: This study aims to assess the general population's attitude and knowledge about the symptoms, signs, and complications of geriatric depression to improve the quality of life of the elderly.

Method: This is a cross-sectional study, using a convenience sample of 2,320 participants, between March to September 2022. However, due to age, nationality, and incomplete responses that did not meet our inclusion criteria, 629 participants were excluded, and the sample size narrowed to 1,691. A structured questionnaire was designed to collect data based on a comprehensive literature review. An online survey was distributed to Saudi citizens. The participants were between 18 and 50, both women and men and only Saudi nationals were included. This age group was selected as a convenient, purposeful sample, assuming that this portion of the population will be able to be enrolled in an electronic survey in addition to their direct contact with elderlies as possible caregivers. All analyses were performed using IBM SPSS Statistics software for Windows, version 26.0 (IBM Corp., Armonk, NY). The data were analyzed using a nonparametric test because they were not normally distributed.

Results: The study comprised 1,691 participants from diverse regions of Saudi Arabia, with a significant gender disparity observed, including 1,249 females (73.9%) and 442 males (26.1%). The majority of our participants were between 18-29 years, accounting for 55.2% of the sample. Descriptive statistics revealed prevalent beliefs among participants regarding geriatric depression. Notably, 35.1% strongly agreed and 19.3% agreed that depression affects individuals of particular ages, while a significant portion (47.1%) disagreed with the statement that depression in the elderly is a health problem. Additionally, 33.9% disagreed that geriatric depression can lead to suicide, and 33.8% believed it can be prevented. Analysis of actions and behaviors highlighted avoidance tendencies, with only 1.5% of the population strongly agreeing to treat a family member with geriatric depression and a majority (63.7%) avoiding interaction due to fear of harm, including 29.5% who strongly agreed and 34.2% who agreed. Gender differences were evident in emotional responses and knowledge levels, with females exhibiting higher emotional responses (mean score 15.63±2.92) and males displaying greater knowledge (mean score 14.90±3.36).

Conclusion: In this study, we investigated the knowledge and attitudes of Saudis toward depression in the elderly. Findings revealed an inadequate understanding of geriatric depression, with many not recognizing it as a health condition and underestimating its severity. Negative emotions and behaviors, such as shame and reluctance to provide support, were prevalent. Gender, education level, and region influenced attitudes and knowledge. These results underscore the need for targeted interventions to raise awareness and challenge the stigma surrounding geriatric depression in Saudi Arabia.

## Introduction

Depression is a leading cause of disability across the world, and it contributes significantly to the overall burden of any disease [1]. According to the World Health Organization (WHO), 350 million people suffer from depression, with over 800,000 individuals committing suicide each year [2]. Between 2000 and 2025, the number of people over 60 will increase by 300% globally [3]. According to Khraif et al. (2015), aging in the Arab world will grow significantly in 2050 [4]. Furthermore, as noted by the United Nations (2014), the population of Saudi Arabians aged 65 and over will continue to expand, and this older group will account for 18.4% of the country's total population in 2050. As the population of the elderly continues to grow as a result of longer lifespans, there will be an increase in the number of individuals in this population suffering from undiagnosed geriatric depression [5]. According to the WHO, the world's elderly population has a point prevalence of depressive disorders ranging from 10% to 20%, depending on cultural circumstances [6].

Per the Centers for Disease Control and Prevention (CDC), depression is a real and treatable medical disease. The hallmark of depression in the elderly is characterized by its comorbidity with medical conditions [7]. Also, according to the CDC, around 80% of older people have at least one chronic health problem, and 50% have two or more chronic health concerns that can be caused by depression [8]. Moreover, the CDC indicated that depression rates among the elderly with chronic conditions and those with reduced capabilities are higher than in the general population, which might exacerbate the situation by reducing quality of life, increasing healthcare costs, utilizing critical healthcare resources, increasing mortality, and doubling the rate of suicide compared to the general population [7]. Studies show that the incidence of depression among the elderly is much higher in Arab countries, with 24.3% in Jordan, 16.9% in Oman, 38.9% in Iraq, 38.7% in Egypt, 25% in Kuwait, and 17.5% in Saudi Arabia [9]. This is due to the misdiagnosis of geriatric depression and a lack of community knowledge about the importance of early depression screening to improve therapeutic outcomes and quality of life [9].

Nowadays, attitudes toward aging among Saudi older adults appear to have changed. Across decades, Saudis tend to show a positive attitude toward aging among older adults. The history of honoring, appreciating, and caring for older individuals is deeply rooted in Saudi Arabia; it is the basis for Saudi society's positive attitude about aging. However, the growth of urbanization, the enormous outflow of labor in Saudi Arabia, and life concerns have forced the new generation to live apart from their elderly relatives. This lifestyle often presents significant obstacles and problems for adult children in providing appropriate care for their elderly parents, and the elderly become a burden. Based on that, it is important to spread knowledge about geriatric depression in order to start early screening, social support interventions, early identification, and management of depressive symptoms to increase the population's well-being. Therefore, this study aims to assess the general population's attitude and knowledge about the symptoms, signs, and complications of geriatric depression in order to improve the quality of life of the elderly.

## Materials and methods

Study design and subjects

This is a cross-sectional, analytical observational study using a non-probability convenience sample that involved 2,320 participants from the adult Saudi population between March 2022 and September 2022. Initially, a total of 2,320 individuals filled up the questionnaire. However, due to age, nationality, and incomplete responses that did not meet our inclusion criteria, 629 participants were excluded, and the sample size narrowed to 1,691. In this research, we included only participants in the age group of 18-50, Saudi nationals of both genders, including five regions in Saudi Arabia: Middle, East, West, North, and South. This age group was selected as a convenient, purposeful sample, assuming that this portion of the population will be able to be enrolled in an electronic survey in addition to their direct contact with elderlies as possible caregivers. The calculation of sample size was done by using the EPI Info statistical package (CDC, Atlanta, GA) for calculating the minimal sample size with a 99% level of confidence (alpha = 0.05) and a power of study of 99% (beta = 0.05). The minimal sample size needed for the current study was 1,500.

Data collection methods, instruments used, and measurements

Based on a comprehensive literature review of knowledge and attitudes regarding geriatric depression in different countries, a structured questionnaire was designed to collect data. The questionnaire was first written in English, then translated into Arabic, and then it was translated back into English. The first part of the questionnaire measured independent demographic variables, including age, gender, citizenship, education level, monthly income, marital status, and place of residence. In section II, a 25-item Likert scale ranging from "strongly agree" to "strongly disagree" was used to assess knowledge of geriatric depression symptoms, causes, and treatment. The next set of 16 Likert items in section III assessed respondents' attitudes regarding geriatric depression.

Validity and reliability

The validity of the questionnaire content was checked, and a pilot study was conducted on 20 respondents from our sample. The pilot study assessed respondents' interpretation of questions and identified issues related to clarity, respondent burden, and transition. Cronbach's alpha test, which is used to assess inter-item reliability, showed a coefficient of 0.862.

Statistical analysis

The study's aim was mainly descriptive. However, we suspected a priori that self-reported attitudes and knowledge of the general population may be related to age, education, employment status, gender, and residency. Data were collected, coded, and analyzed using IBM SPSS Statistics software for Windows, version 26.0 (IBM Corp., Armonk, NY) under Microsoft Windows (Microsoft Corp., Redmond, WA). Simple descriptive analysis was done, followed by inferential statistics using nonparametric tests whenever needed for not normally distributed data, and comparisons were assessed by the independent samples, Mann-Whitney U test, and Kruskal-Wallis test. Statistical significance was set at p < 0.05.

Ethical consideration

The study has ethical clearance from the institutional research ethical committee at Princess Nourah bint Abdulrahman University, Riyadh, Kingdom of Saudi Arabia (KSA) with the approval number HAP-01-R-059. Written consent was obtained from participants who received the online questionnaire. Furthermore, all subjects were informed that participation in the study is voluntary and that the data collected were anonymous, confidential, and will be restricted for this study only.

## Results

The participants' sociodemographic characteristics are presented in Table 1, which shows that 1,691 participants from different regions of Saudi Arabia participated in the study. Nearly three-quarters of respondents were female (1,249, 73.9%), while male respondents were 442 (26.1%). The majority of respondents were in the 18-29-year age group. Most of the respondents had a high level of education; 695 (41.1%) were university students, and 585 (34.6%) were graduates. Almost half of the respondents (787, 46.4%) had enough monthly income, and 998 (59.0%) respondents were single.

**Table 1:** Demographic data of the participants n: number of subjects

Variables	Frequency (%) (n=1691)
Gender	
Male	442 (26.1)
Female	1249 (73.9)
Age (years)	
18-29	1067 (63.1)
30-39	290 (27.2)
40-50	334 (29.8)
Level of education	
Illiterate	18 (1.1)
School	302 (17.9)
University students	695 (41.1)
University graduate	585 (34.6)
Postgraduate	91 (5.4)
Employment status	
Students	787 (46.5)
Healthcare worker	168 (9.9)
Not a healthcare worker	362 (21.4)
Unemployed	217 (12.8)
Housewife	157 (9.3)
Income	
Enough	784 (46.4)
Enough and save	300 (17.7)
Not enough	418 (24.7)
Not enough; have debt	189 (11.2)
Marital status	
Single	998 (59.0)
Married	636 (37.6)
Divorced	40 (2.4)
Widow/Widower	17 (1.0)
Region of residency	
South	328 (19.4)
West	322 (19.0)
East	367 (21.7)
North	286 (16.9)
Middle	388 (22.9)

Table 2 illustrates the general knowledge of the participants regarding geriatric depression. Almost half of the participants strongly agreed (35.1%) and agreed (19.3%) that depression affects individuals of particular ages. A plurality (47.1%) of respondents disagreed with the statement that depression in the elderly is considered a health problem. The majority of participants (33.9%) disagreed that geriatric depression can lead to suicide. There was a strong belief among 33.8% of participants that geriatric depression can be prevented.

**Table 2:** General knowledge of the participants regarding geriatric depression n: number of subjects Scores were awarded as follows: a score of 1 for "strongly disagree" to a score of 5 for "strongly agree." The mean and standard deviation of each response were calculated by coding responses according to their respective scores (1–5).

Variables	Strongly disagree		Disagree		Neutral		Agree		Strongly agree		Mean ± SD
	N	(%)	N	(%)	N	(%)	N	(%)	N	(%)	
Depression affects people of a particular age group	121	7.2	290	17.1	359	21.2	594	35.1	327	19.3	3.42 ± 1.18
Becoming depressed is a natural part of old age	112	6.6	505	29.9	501	29.6	433	25.6	140	8.3	2.99 ± 1.07
Geriatric depression is considered a health problem	349	20.6	797	47.1	336	19.9	158	9.3	51	3	2.27 ± 0.98
Geriatric depression can lead to suicide and suicide attempts	269	15.9	510	30.2	460	27.2	354	20.9	98	5.8	2.71 ± 1.13
Older people with depression are dangerous to themselves and others	283	16.7	574	33.9	484	28.6	266	15.7	84	5	2.58 ± 1.09
Geriatric depression cannot be prevented.	96	5.7	292	17.3	449	26.6	571	33.8	283	16.7	3.39 ± 1.12
Total											17.35 ± 3.72

Table 3 describes the emotions of participants regarding geriatric depression. More than half of the participants strongly agreed and agreed (35.5% and 32.3%, respectively) with being ashamed of having an elderly family member with depression; 41.3% strongly disagreed and 41.6% disagreed with feeling sympathy for old people with depression; and 32.6% strongly agreed with feeling afraid of being close to old people with depression because there is a risk of contracting the same illness.

**Table 3:** Emotions of participants regarding geriatric depression n: number of subjects Scores were awarded as follows: a score of 1 for "strongly disagree" to a score of 5 for "strongly agree." The mean and standard deviation of each response were calculated by coding responses according to their respective scores (1–5).

Variables	Strongly disagree		Disagree		Neutral		Agree		Strongly agree		Mean ± SD
	N	(%)	N	(%)	N	(%)	N	(%)	N	(%)	
I feel comfortable dealing with the needs of old depressed people.	210	12.4	549	32.5	600	35.5	263	15.6	69	4.1	2.66 ± 1.01
I am ashamed of having an elderly family member who has depression.	63	3.7	210	12.4	272	16.1	546	32.3	600	35.5	3.83 ± 1.14
I sympathize with old people with depression.	698	41.3	704	41.6	218	12.9	53	3.1	18	1.1	1.81 ± 0.85
I am afraid of being close to old people with depression because they can result in harm.	95	5.6	224	13.2	450	26.6	556	32.9	366	21.6	3.52 ± 1.13
I am afraid of being close to old people with depression because there is a risk of contracting the same illness.	96	5.7	242	14.3	353	20.9	448	26.5	552	32.6	3.66 ± 1.22
Total											15.48 ± 2.95

Table 4 shows actions and behaviors toward geriatric depression. Only 1.5% of the population strongly agreed to treat a family member with geriatric depression. More than half of the participants avoided the elderly with depression because it can result in harm (29.5% strongly agreed and 34.2% agreed).

**Table 4:** Actions and behavior towards geriatric depression n: number of subjects Scores were awarded as follows: a score of 1 for "strongly disagree" to a score of 5 for "strongly agree." The mean and standard deviation of each response were calculated by coding responses according to their respective scores (1–5).

Variables	Strongly disagree		Disagree		Neutral		Agree			Strongly agree		Mean ± SD
	N	(%)	N	(%)	N	(%)	N	(%)		N	(%)	
I seek help for my related elderly if they show signs of depression.	538	31.8	756	44.7	266	15.7	96	5.7		35	2.1	2.01 ± 0.94
I treat my family member if he/ she gets geriatric depression.	591	34.9	715	42.3	278	16.4	82	4.8		25	1.5	1.96 ± 0.91
I'm capable of supporting my family member if he/ she gets geriatric depression.	509	30.1	682	40.3	387	22.9	92	5.4		21	1.2	2.07 ± 0.92
I avoid old people with depression because it can result in harm.	77	4.6	200	11.8	336	19.9	579	34.2		499	29.5	3.72 ± 1.14
I share my house with an elderly family member who has depression.	349	20.6	635	37.6	487	28.8	151	8.9		69	4.1	2.38 ± 1.03
I deal with the needs of old depressed people.	277	16.4	613	36.3	599	35.4	162	9.6		40	2.4	2.45 ± 0.95
Total												14.60 ± 3.36

Table 5 shows knowledge about the concept of geriatric depression. Approximately half of the participants (49.4%) disagreed that geriatric depression can affect social and functional performance. Only 7.2% strongly disagreed that keeping old people who suffer from depression in the hospital keeps the community safer.

**Table 5:** Knowledge about the concept of geriatric depression n: number of subjects Scores were awarded as follows: a score of 1 for "strongly disagree" to a score of 5 for "strongly agree." The mean and standard deviation of each response were calculated by coding responses according to their respective scores (1–5).

Variables	Strongly disagree		Disagree		Neutral		Agree		Strongly agree		Mean ± SD
	N	(%)	N	(%)	N	(%)	N	(%)	N	(%)	
Depression is a normal reaction to changes in old age	156	9.2	443	26.2	502	29.7	407	24.1	183	10.8	3.01 ± 1.14
Geriatric depression can affect social and functional performance	451	26.7	836	49.4	312	18.5	69	4.1	23	1.4	2.04 ± 0.85
Geriatric depression may be a manifestation of medical disease (e.g., cancer, Parkinson's, and Alzheimer's)	311	18.4	654	38.7	473	28	205	12.1	48	2.8	2.42 ± 1.01
Keeping old people who suffer from depression in the hospital keeps the community safer	122	7.2	256	15.1	502	29.7	513	30.3	298	17.6	3.36 ± 1.14
Older people who complain of feeling down are often just looking for attention	281	16.6	523	30.9	432	25.5	284	16.8	171	10.1	2.73 ± 1.21
Total											13.56 ± 3.02

Table 6 demonstrates the participants' knowledge about the symptoms of geriatric depression; 50.7% of the participants disagreed that most people with geriatric depression deal with sleeping problems. Only 0.8% of them strongly agreed that people with geriatric depression often feel helpless and worthless.

**Table 6:** Knowledge about the symptoms of geriatric depression n: number of subjects Scores were awarded as follows: a score of 1 for "strongly disagree" to a score of 5 for "strongly agree." The mean and standard deviation of each response were calculated by coding responses according to their respective scores (1–5).

Variables	Strongly disagree		Disagree		Neutral		Agree		Strongly agree		Mean ± SD
	N	(%)	N	(%)	N	(%)	N	(%)	N	(%)	
People with geriatric depression suffer from changes in appetite and bowel movements	413	24.4	822	48.6	361	21.3	66	3.9	29	1.7	2.10 ± 0.87
Difficulty concentrating, restlessness, and feeling overly guilty about things are symptoms of geriatric depression	356	21.1	819	48.4	431	25.5	75	4.4	10	0.6	2.15 ± 0.82
Most people with geriatric depression deal with sleeping problems	397	23.5	858	50.7	363	21.5	63	3.7	10	0.6	2.07 ± 0.80
Loss of interest in things previously enjoyed is a sign of geriatric depression	435	25.7	780	46.1	374	22.1	79	4.7	23	1.4	2.10 ± 0.88
Older people with depression often report physical aches and pains rather than sadness	307	18.2	615	36.4	597	35.3	151	8.9	21	1.2	2.39 ± 0.92
People with geriatric depression often feel helpless and worthless	466	27.6	837	49.5	327	19.3	48	2.8	13	0.8	2.00 ± 0.80
Total											12.80 ± 3.81

Regarding the knowledge about the causes of geriatric depression, we found that 35.8% of participants disagreed with the statement that genetics is one of the possible causes of geriatric depression, and half of the participants disagreed with the idea that marital or relationship problems can lead to geriatric depression. Additionally, 32.1% of participants disagreed with the idea that a low educational level is a risk factor for geriatric depression, and 45.3% disagreed that loneliness can cause geriatric depression. The statement that stressful life events (e.g., the death of a loved one) may lead to geriatric depression was disagreed with by 44.6% of participants, while 41.3% disagreed that physical conditions (e.g., stroke, diabetes, heart diseases, cancer, and dementia) are possible causes that end in geriatric depression (Table 7).

**Table 7:** Knowledge about the causes of geriatric depression n: number of subjects Scores were awarded as follows: a score of 1 for "strongly disagree" to a score of 5 for "strongly agree." The mean and standard deviation of each response were calculated by coding responses according to their respective scores (1–5).

Variables	Strongly disagree		Disagree		Neutral		Agree		Strongly agree		Mean ± SD
	N	(%)	N	(%)	N	(%)	N	(%)	N	(%)	
Genetics is one of the possible causes of geriatric depression	182	10.8	405	24	606	35.8	348	20.6	150	8.9	2.93 ± 1.10
Marital/relationship problems can result in geriatric depression	347	20.5	845	50	384	22.7	96	5.7	19	1.1	2.17 ± 0.85
Low educational level is one of the risk factors for geriatric depression	206	12.2	500	29.6	543	32.1	331	19.6	111	6.6	2.79 ± 1.09
Loneliness (e.g., being single, unmarried, divorced, or a widow) can result in geriatric depression	486	28.7	766	45.3	324	19.2	91	5.4	24	1.4	2.05 ± 0.90
Stressful life events (e.g., the death of a loved one) may lead to geriatric depression	626	37	754	44.6	243	14.4	49	2.9	19	1.1	1.87 ± 0.84
Physical conditions (e.g., stroke, diabetes, heart disease, cancer, and dementia) are possible causes that end with geriatric depression	457	27	698	41.3	431	25.5	79	4.7	26	1.5	2.12 ± 0.91
Total											13.92 ± 3.71

Table 8 shows participants' knowledge of the management of geriatric depression. Among them, 21.9% of the participants strongly agreed that there is little that can be done to help an older person with depression. On the other hand, 39.1% strongly agreed with the statement, ‘‘if the elderly I am caring for tells me that she/he is depressed, it is best to leave them alone’’. Furthermore, responses to the statement ‘‘talking with a depressed elderly about their condition may make things worse’’ (30.8%) were neutral about this statement. Most of the participants disagreed that solving one’s social problems (e.g., poverty and family problems) can overcome geriatric depression; 40.5% of the participants strongly disagreed with the idea that strong social support from relatives and friends can help older people with depression overcome their illness. Regarding the statement "geriatric depression can be treated with pharmacological methods," we found that 39.4% of the participants disagreed, and 45.2% disagreed that psychological treatment, such as talking to a therapist or counselor, is useful. Also, 43.1% disagreed that geriatric depression can be overcome with stress management techniques.

**Table 8:** Knowledge about the management of geriatric depression n: number of subjects Scores were awarded as follows: a score of 1 for "strongly disagree" to a score of 5 for "strongly agree." The mean and standard deviation of each response were calculated by coding responses according to their respective scores (1–5).

Variables	Strongly disagree		Disagree		Neutral		Agree		Strongly agree		Mean ± SD
	N	(%)	N	(%)	N	(%)	N	(%)	N	(%)	
There is little that can be done to help an older person with depression	98	5.8	201	11.9	367	21.7	655	38.7	370	21.9	3.59 ± 1.12
If the elderly I am caring for tells me that she/he is depressed, it is best to leave them alone	68	4	178	10.5	263	15.6	521	30.8	661	39.1	3.90 ± 1.14
Talking with a depressed elderly about their condition may make things worse	109	6.4	350	20.7	521	30.8	469	27.7	242	14.3	3.23 ± 1.12
Solving one’s social problems (e.g., poverty and family problems) can overcome geriatric depression	354	20.9	787	46.5	419	24.8	90	5.3	41	2.4	2.22 ± 0.91
Older people with depression can overcome their illness with strong social support from relatives and friends	685	40.5	650	38.4	271	16	56	3.3	29	1.7	1.87 ± 0.91
Geriatric depression can be treated with pharmacological methods	262	15.5	666	39.4	612	36.2	116	6.9	35	2.1	2.41 ± 0.90
Psychological treatment such as talking to a therapist, or a counselor is useful	560	33.1	765	45.2	275	16.3	66	3.9	25	1.5	1.95 ± 0.88
Geriatric depression can be overcome with stress management techniques	338	20	729	43.1	531	31.4	71	4.2	22	1.3	2.24 ± 0.86
Total											21.40 ± 3.97

Table 9 compares participants' attitudes and knowledge regarding geriatric depression based on their gender. Females had greater emotions (15.63 ± 2.92) with a p-value of <0.001, whereas men had a higher mean regarding action and behavior (14.90 ± 3.36) with a p-value of 0.035 and a higher mean regarding knowledge of the concept of geriatric depression (13.90 ±2.93) with a p-value of 0.035, as well as a higher mean regarding knowledge of the symptoms of geriatric depression (13.60 ± 3.80) with a p-value of <0.001.

**Table 9:** A comparison of participants' attitudes and knowledge regarding geriatric depression based on their gender The p-value is based on the independent sample Mann-Whitney U test. Scores were awarded as follows: a score of 1 for "strongly disagree" to a score of 5 for "strongly agree." The mean and standard deviation of each response were calculated by coding responses according to their respective scores (1–5).

Variables	Male (442)		Female (1249)		P-value
						Mean ± SD		Mean ± SD	
	Attitude					
Knowledge	17.52 ± 4.073		17.29 ± 3.59		0.409
Emotions	15.07 ± 2.97		15.63 ± 2.92		<0.001*
Action and behaviors	14.90 ± 3.36		14.49 ± 3.35		0.035*
Knowledge					
Knowledge about the concept of geriatric depression	13.90 ± 2.93		13.44 ± 3.05		0.035*
Knowledge about the symptoms of geriatric depression	13.60 ± 3.80		12.52 ± 3.78		<0.001*
Knowledge about the causes of geriatric depression	14.22 ± 3.63		13.82 ± 3.73		0.064
Knowledge about the management of geriatric depression	21.48 ± 3.97		21.38 ± 3.97		0.811

Table 10 shows a comparison of participants' attitudes and knowledge regarding geriatric depression based on their region of residence in Saudi Arabia. Regarding the attitude section, participants from the Middle region had the highest mean in knowledge (17.65 ± 3.53) with a p-value of 0.003, while participants from the North region had the highest mean in emotions (15.76 ± 3.00) with a p-value of 0.001, and the participants from the West region had the highest mean in action and behaviors (15.04 ±3.31) with a p-value of 0.001.

**Table 10:** A comparison of participants' attitudes and knowledge regarding geriatric depression based on their region of residence in Saudi Arabia The p-value is based on the Kruskal-Wallis test. Scores were awarded as follows: a score of 1 for "strongly disagree" to a score of 5 for "strongly agree." The mean and standard deviation of each response were calculated by coding responses according to their respective scores (1–5).

Variables	South region (328)	West region (322)	East region (367)	North region (286)	Middle region (388)	P-value
	Mean ± SD	Mean ± SD	Mean ± SD	Mean ± SD	Mean ± SD	
Attitude						
Knowledge	17.51 ± 3.49	16.77 ± 3.46	17.48 ± 3.67	17.26 ± 4.44	17.65 ± 3.53	0.003*
Emotions	15.83 ± 2.76	14.58 ± 3.25	15.57 ± 2.71	15.76 ± 3.00	15.64 ± 2.87	<0.001*
Action and behaviors	14.03 ± 3.41	15.04 ± 3.31	14.94 ± 2.95	14.30 ± 3.71	14.62 ± 3.38	<0.001*
Knowledge						
knowledge about the concept of geriatric depression	13.45 ± 2.90	13.35 ± 2.95	13.69 ± 2.71	12.97 ± 3.69	14.13 ± 2.82	<0.001*
Knowledge about the symptoms of geriatric depression	12.72 ± 3.59	13.59 ± 3.59	12.70 ± 3.32	11.83 ± 4.54	13.02 ± 3.88	<0.001*
Knowledge about the causes of geriatric depression	13.92 ± 3.64	14.59 ± 3.45	14.05 ± 3.51	12.84 ± 4.27	14.05 ± 3.56	<0.001*
Knowledge about the management	21.89 ± 4.33	20.97 ± 3.87	21.40 ± 3.56	20.96 ± 4.27	21.68 ± 3.79	<0.011*

Regarding the knowledge section, the Middle region had the highest mean for knowledge of the concept of geriatric depression (17.65 ± 3.53) with a p-value of 0.003, whereas the West region had the highest mean for knowledge of the symptoms and causes (13.59 ± 3.59 and 14.59 ± 3.45) with a p-value of 0.001, while the South region had the greatest mean in regard to treatment (21.89 ± 4.33) with a p-value of 0.011.

Table 11 illustrates that there’s a difference between age groups in the knowledge and emotions in the attitude section, which shows that the participants between 40 and 50 years old had higher knowledge (p-value <0.001), but participants between 18 and 29 years old had higher emotions regarding the elderly with depression (p-value <0.001), which is statistically significant. Participants aged 40 to 50 had higher knowledge regarding the concept, symptoms, causes, and management (with p-values of 0.016, 0.019, 0.006, and 0.012, respectively), which is statistically significant.

**Table 11:** A comparison of participants' attitudes and knowledge regarding geriatric depression based on their age The p-value is based on the independent sample Mann-Whitney U test. Scores were awarded as follows: a score of 1 for "strongly disagree" to a score of 5 for "strongly agree." The mean and standard deviation of each response were calculated by coding responses according to their respective scores (1–5).

Variables	Age			P-value
	18-29 years old (1067)	30-39 years old (290)	40-50 years old (334)	
	Mean ± SD	Mean ± SD	Mean ± SD	
Attitude				
Knowledge	17.12 ± 3.58	17.42 ± 4.02	18.02 ± 3.81	<0.001*
Emotions	15.65 ± 3.02	15.03 ± 2.98	15.35 ± 2.64	<0.001*
Action and behaviors	14.49 ± 3.48	14.89 ± 3.29	14.68 ± 3.00	0.097
Knowledge				
Knowledge about the concept of geriatric depression	13.60 ± 3.11	13.22 ± 2.98	13.72 ± 2.75	0.016*
Knowledge about the symptoms of geriatric depression	12.62 ± 3.87	12.85 ± 3.96	13.32 ± 3.43	0.019*
Knowledge about the causes of geriatric depression	13.74 ± 3.84	13.94 ± 3.70	14.51± 3.20	0.006*
Knowledge about the management of geriatric depression	21.35 ± 3.98	21.05 ± 4.02	21.90 ± 3.84	0.012*

Table 12 shows a comparison of attitude and knowledge regarding geriatric depression according to the participants' educational level. There is a recognizable difference between the educational levels in the attitude section regarding knowledge and emotions. Postgraduates had the greatest mean in terms of knowledge, with 17.86 ± 3.55, (p-value <0.001). On the other hand, university graduates had the highest mean on the emotions section with 15.56 ± 2.66 (p-value <0.001). Furthermore, the illiterate group demonstrated a higher mean regarding knowledge about the causes of geriatric depression, 14.66 ± 3.59 (p-value of 0.006). In contrast, university students show the lowest mean with 13.58 ± 3.90 (p-value of 0.006).

**Table 12:** A comparison of participants' attitudes and knowledge regarding geriatric depression based on their educational level. The p-value is based on the Kruskal-Wallis test. Scores were awarded as follows: a score of 1 for "strongly disagree" to a score of 5 for "strongly agree." The mean and standard deviation of each response were calculated by coding responses according to their respective scores (1–5).

Variables	Illiterate (18)	School (302)	University students (695)	University graduate (585)	Postgraduate (91)	P-value
	Mean ± SD	Mean ± SD	Mean ± SD	Mean ± SD	Mean ± SD	
Attitude						
Knowledge	15.00 ± 3.61	17.05 ± 3.80	17.09 ± 3.62	17.82 ± 3.75	17.86 ± 3.55	<0.001 *
Emotions	13.27 ± 2.44	15.18 ± 3.17	15.63 ± 3.06	15.56 ± 2.66	15.32 ± 2.92	<0.001*
Action and behaviors	15.50 ± 4.39	14.59 ± 3.37	14.51 ± 3.62	14.65 ± 3.02	14.80 ± 3.18	0.487
Knowledge						
Knowledge about the concept of geriatric depression	12.00 ± 2.95	13.41 ± 3.14	13.53 ± 3.16	13.68 ± 2.75	13.70 ± 3.14	0.162
Knowledge about the symptoms of geriatric depression	13.94 ± 4.13	13.21 ± 3.82	12.49 ± 3.92	12.89 ± 3.61	13.00 ± 4.04	0.049
Knowledge about the causes of geriatric depression	14.66 ± 3.59	14.46 ± 3.71	13.58 ± 3.90	14.07 ± 3.41	13.64 ± 3.86	0.006
Knowledge about the management of geriatric depression	20.61 5.15	21.21 ± 3.99	21.22 ± 4.19	21.65 ± 3.56	22.03 ± 4.24	0.502

Table 13 compares the participants' attitudes and knowledge according to their employment status. The attitude section (knowledge and emotions) was significant, as the housewives scored the highest mean (p-value of <0.001). On the other hand, emotions were highly scored by students (p-value of <0.001). Regarding knowledge, only the knowledge about the causes of geriatric depression was significant, and the higher mean was among housewives (p-value of 0.015).

**Table 13:** A comparison of participants' attitudes and knowledge regarding geriatric depression based on their employment status The p-value is based on the Kruskal-Wallis test. Scores were awarded as follows: a score of 1 for "strongly disagree" to a score of 5 for "strongly agree." The mean and standard deviation of each response were calculated by coding responses according to their respective scores (1–5).

Variables	Students (787)	Healthcare worker (168)	Not a healthcare worker (362)	Unemployed (217)	Housewife (157)	P-value
	Mean ± SD	Mean ± SD	Mean ± SD	Mean ± SD	Mean ± SD	
Attitude						
Knowledge	17.08 ± 3.53	16.76 ± 3.78	17.59 ± 3.95	17.34 ± 3.90	17.98 ± 3.55	<0.001*
Emotions	15.65 ± 3.08	15.25 ± 3.11	15.34 ± 2.77	15.41 ± 2.98	15.29 ± 3.37	0.019*
Action and behaviors	14.48 ± 3.52	14.91 ± 3.50	14.76 ± 3.17	14.43 ± 3.34	14.70 ± 2.74	0.314*
Knowledge						
Knowledge about the concept of geriatric depression	13.52 ± 3.22	13.83 ± 3.03	13.62 ± 3.73	13.71 ± 3.07	13.09 ± 2.46	0.149*
Knowledge about the symptoms of geriatric depression	12.49 ± 3.94	12.82 ± 3.73	13.06 ± 3.78	13.32 ± 3.72	13.04± 3.35	0.063*
Knowledge about the causes of geriatric depression	13.63 ± 3.88	13.64 ± 3.59	14.16 ± 3.61	14.37 ± 3.53	14.55 ± 3.27	0.015*
Knowledge about the management of geriatric depression	21.25 ± 4.04	21.49 ± 3.79	21.52 ± 3.84	21.78 ± 4.06	21.33 ± 3.95	0.927

## Discussion

This study aimed to investigate the knowledge and attitudes of Saudis toward depression in the elderly. More than 1,691 participants participated in our survey, most of whom were between the ages of 18 and 29, and around three-quarters of them were females. Additionally, most of them had a university degree and did not work in the healthcare sector.

The current study results demonstrated that participants had inadequate knowledge about the general concept, causes, symptoms, and management of geriatric depression. This might reflect the low level of awareness among the Saudi population about this condition.

In our survey, participants' knowledge of geriatric depression was generally insufficient, with the majority (47.1%) disagreeing that depression in the elderly is considered a health condition. This might be related to the stigma of depression and the perception that having a weak personality leads to depression. In a 2003 study comparing the stigma attached to depression in Japanese and Australians, 45.4% of Japanese held the attitude that "the problem is a sign of personal weakness" and 40.2% believed that "the problem is not a medical illness, while only 13.4% and 14.6% of Australians, respectively, had this attitude [10]. Similarly, a survey of primary healthcare providers in Cameroon found that 67.3% of respondents disagreed that depression in the elderly is considered a health problem [11]. Furthermore, we discovered that the majority of participants (33.9%) did not believe that geriatric depression may lead to suicide. In contrast, a different study in Cameroon showed that 92.9% of respondents denied that geriatric depression may lead to suicide [11].

The lack of knowledge about geriatric depression and its nature highlights the negative emotions and behaviors experienced by elderly individuals with depression. The current study reveals that 35.5% strongly agreed, and 32.3% agreed that having an elderly family member with depression is a source of shame. While a study in Shanghai showed a lower percentage (10.22%) of illiterate people, 9.68% of literate people agreed that having dementia is shameful [12]. In addition, a study done in the KSA recently showed that 62.7% of people are willing to make friendships with depressed individuals [13].

More than half of the participants showed agreement to being afraid of being close to an elderly person with depression because it can result in harm; 32.9% agreed and 21.6% strongly agreed. A study done in the United Arab Emirates (UAE) showed that 34.9% are afraid to be close to individuals with depression and schizophrenia because they can result in harm [3]. A study released in Italy showed that a lower percentage (27%) said that individuals suffering from depression are dangerous to others [4]. In addition, about one-third of respondents (32.6%) strongly agreed with being afraid to be close to old people with depression because there is a risk of contracting the same illness, which can be explained by a lack of knowledge about the nature of the disease. While in Italy, 16% agreed that contact with depressed individuals would expose them to contracting the same illness [14].

Surprisingly, we found that a greater number of respondents are not willing to treat their family members if they are diagnosed with geriatric depression (34.9% strongly disagreed and 42.3% disagreed). This illustrates the lack of knowledge about methods of treating geriatric depression. In contrast, a study in Cameroon reported that 78.4% of participants would send depressed patients for counseling or medical treatment, and 21% of participants would send them home for rituals or traditional healers [1]. These results demonstrate a crucial need to correct negative attitudes and promote positive images toward geriatric depression and the need for management. Moreover, 85.3% of Arab American Muslims think that depression is considered an illness and requires intervention [15].

Furthermore, we found that 29.5% strongly agreed and 34.2% agreed to avoid the elderly with depression because it can result in harm. This indicates a high level of social distance toward people suffering from geriatric depression due to the belief that they are more aggressive than others. A study established in the UAE with a sample size of 430 showed that 45.6% will avoid the elderly with depression because they have disturbing and negative thoughts [16].

Approximately half of the participants thought that depression would not affect the social and functional performance of the affected elderly. This emphasizes the inadequacy of knowledge regarding the concept that geriatric depression is a disease that can affect one’s functioning in life. On the contrary, a previous investigation regarding general depression in Italy showed that about one-third of their sample thought that depression could be a disabling condition. It was considered prejudice towards depression and people with depression in their study [14].

Moreover, a large number of the participants agreed and strongly agreed (30.3% and 17.6%, respectively) that keeping old people who suffer from depression in the hospital keeps the community safer. These findings indicate a significant amount of social distancing toward the elderly who suffer from depression. The thought of isolating them from the community can support the previous results that they might be harmful and should be avoided. This is similar to a study done in the UAE, which showed that the majority of their sample stated that individuals with depression and schizophrenia are unpredictable and can result in harm, while only 34.9% were not afraid of being close to them [16]. On the other hand, a recent study done in Saudi Arabia investigating the degree of public awareness, beliefs, and attitudes regarding major depression showed that 62.7% of the participants were willing to form friendships with depressed individuals and 63.9% were willing to work with them [13]. This shows that the population might empathize more with a younger, depressed individual than an older one.

There is a noted lack of knowledge about the symptoms of geriatric depression as well. Analysis of the items that investigated knowledge about geriatric depression symptoms showed that only a small number were able to recognize sleep problems (0.6%), change in appetite (1.7%), difficulty concentrating, guilt feeling (0.6%), loss of interest (1.4%), and feeling helpless and worthless (0.8%) as symptoms of geriatric depression. In contrast, other studies addressing knowledge regarding general depression showed great knowledge about these symptoms (85.4%) [13]. The possible explanation for this inconsistency is that depression symptoms might be familiar to the population, but when these symptoms are presented in the elderly, they might be confused with normal aging manifestations.

In general, our results showed that there is a lack of knowledge about the causes of geriatric depression. It may be because of poor education and awareness regarding the causes of geriatric depression among the general population. Regarding knowledge about the causes of geriatric depression, we found that 45.3% disagreed that loneliness can result in geriatric depression. For the statement "stressful life events (e.g., death of a loved one) may lead to geriatric depression," 44.6% of the participants disagreed with this statement. In a study done in Italy, they found many respondents associate depression with a specific life stressor such as serious illness (86%), divorce or separation (84%), and mourning for a friend or close family member (82%) [14].

Regarding the knowledge of the management of geriatric depression, we found that 39.4% of the participants disagreed that depression can be treated with pharmacological methods, while 45.2% disagreed that psychological treatment such as talking to a therapist or a counselor is useful. In a study done in Aseer, Saudi Arabia, on primary care physicians, they found that 94.2% had a positive attitude toward the management of geriatric depression [17]. Additionally, in another study done in Saudi Arabia regarding public awareness, beliefs, and attitudes toward depressive disorders among 1,188 participants, they found that awareness regarding the risk factors (12.5%) and treatment approaches (15.7%) for depression was low [13]. Moreover, a study done in Italy found that about half of the respondents (53%) agreed on the importance of pharmacological treatment. However, many of them also seemed convinced that antidepressant drugs may cause serious side effects (55%) [14]. According to a study conducted in urban Turkey, 89.7% of the public believed that psychological interventions are more effective than pharmacotherapy, while 77.7% believed in the effectiveness of social interventions; 62.1% believed that the medicines used in the treatment of depression are harmful and addictive [8].

Gender differences in attitudes regarding geriatric depression were also discovered. Interestingly, females reported a higher mean (15.63‡2.92) in terms of emotions toward geriatric depression; this may be due to women's higher levels of effectivity, openness, and sensitivity to their feelings [18]. On the other hand, males had a higher mean (14.90 ‡3.36) in terms of action and behavior. This could be explained by men’s restrictive emotionality [19, 20], which is more practical than that of women, whose feelings are more dominant. A previous paper studying youth’s attitudes toward the elderly showed that gender is not an important variable affecting attitudes toward the elderly. The attitudes of males and females within the same age group were very similar [21].

In terms of knowledge, our findings indicated that males reported greater knowledge than females. In contrast, a previous study conducted in KSA showed that 68.9% of female respondents had a good awareness of depression, compared to only 62.8% of male respondents, with a significant gender difference (p = 0.027) [13]. Also, a different study discovered that males were less likely to be familiar with depression and to identify depression symptoms [22].

As expected, higher knowledge about geriatric depression was correlated with a higher educational level. Similarly, a previous study found that participants with higher levels of education were more likely to have greater mental health knowledge [23]. Whereas university students demonstrated a higher level of emotional intelligence (p-value <0.001), this could be attributed to their young age. Evidence shows that positive emotions increase from childhood to young adulthood and then decline around the mid-70s [24]. Surprisingly, the illiterate group exhibited higher knowledge about the causes than the university students (p-value of 0.006). This could be justified by their constant contact for long periods and the close relationship they have with these elderly people. This might give them the opportunity to observe and gain more knowledge about their medical conditions. On the contrary, a study done in China focused on the impact of education on knowledge, attitudes, and stigma related to dementia among illiterate and literate older adults and showed an overall lower percentage of correct answers among the illiterate group [12]. Other investigations showed that those with higher educational levels (65.7%) and those with lower educational levels did not significantly differ in awareness about depression (54.1%; p = 0.061) [13].

In a comparison of attitude and knowledge according to employment status, our study demonstrated that regarding the attitude part (knowledge and emotions), housewives scored the highest knowledge level (p-value of <0.001). This might be attributed to the constant contact of housewives with the elderly population. On the other hand, students showed higher emotional levels (p-value of <0.001). As discussed previously, students might manifest higher emotional levels due to their young age. In terms of actions and behaviors, we discovered that healthcare workers had the highest mean score, although the difference was not significant. This could potentially be attributed to their higher level of knowledge. One of the studies conducted on the attitudes, knowledge, and behavior of family physicians regarding depression in late life revealed that the physicians who responded to the survey were generally aware of alternative presentations of depression in elderly individuals and were well-informed about the duration of treatment with depression medications. The physicians believed that depression medications were equally effective for older patients as they were for younger patients, but they were less optimistic about the effectiveness of psychotherapy [25].

The analysis of the different Saudi regions regarding their knowledge and attitude revealed that the regions with the highest level of knowledge were in the Middle region, followed by the Southern region, the Eastern region, the Northern region, and the Western region. The capital city of Saudi Arabia, Riyadh, is located in the Middle region which has the most developed, civilized, and educated societies; this could be the reason they scored the highest. 

When comparing the different age groups, we found that there is higher knowledge of concepts, symptoms, causes, and management in older age groups with significant p-values in all domains (0.016, 0.019, 0.006, 0.012, respectively). This can be explained by the fact that older people have more experience dealing with the elderly. A different study performed in Saudi Arabia showed similar results with good awareness levels in the older age group (p-value of 0.049) [4].

These findings suggest that our participants are not considering depression in this age group as a real medical condition, and it might be a sequel to other age-related diseases (e.g., Parkinson's disease, Alzheimer's disease, etc.). Due to their lack of understanding of this condition, a considerable number thought these individuals might be dangerous and harmful and therefore should be kept in hospitals. On the other hand, we also observed a lack of knowledge concerning the management and treatment of these individuals. All of this suggests the low awareness of the community in terms of geriatric depression and aging in general. Hence, we need further initiatives to raise community awareness about the mental health of this age group.

Study limitations

A few limitations of the study are acknowledged. Even though the study was conducted using an online self-reporting questionnaire, a good response (n = 1691) was achieved. Moreover, in certain areas of the study, there was no prior research to compare their results with our findings. 

## Conclusions

In conclusion, the study investigated Saudis' knowledge and attitudes regarding depression in the elderly through a survey of 1,691 participants. It found a widespread lack of understanding about geriatric depression, including its causes, symptoms, and management. Many participants did not recognize it as a health condition and underestimated its severity, including the risk of suicide. Negative attitudes such as shame and reluctance to provide support were prevalent, influenced by cultural stigma. Gender, education, region, and age influenced knowledge and attitudes, with women showing higher emotional responses and educated individuals demonstrating better understanding. The findings underscore a critical need for targeted interventions to raise awareness, combat stigma, and improve support for elderly individuals with depression in Saudi Arabia. Further studies may be needed to assess the extent and knowledge regarding geriatric depression in order to help decision-makers in Saudi Arabia implement a national program for the detection and management of geriatric depression.
